# Spatial ecology and conservation of leatherback turtles (*Dermochelys coriacea*) nesting in Bioko, Equatorial Guinea

**DOI:** 10.1371/journal.pone.0286545

**Published:** 2023-06-14

**Authors:** Francesco Garzon, Christian Barrientos, Rigoberto Esono Anvene, Feme Esono Mba, Alejandro Fallabrino, Angela Formia, Brendan J. Godley, Mary K. Gonder, Carolina Martinez Prieto, Jesus Mba Ayetebe, Kristian Metcalfe, David Montgomery, Juan Nsogo, Juan-Cruz Ondo Nze, Earl Possardt, Erick Ross Salazar, Manjula Tiwari, Matthew J. Witt

**Affiliations:** 1 Hatherley Laboratories, Faculty of Health and Life Sciences, University of Exeter, Exeter, Devon, United Kingdom; 2 Wildlife Conservation Society, Candy Bldg, Bata, Equatorial Guinea; 3 Tortugas Marinas de Guinea Ecuatorial (TOMAGE), Instituto Nacional de Desarrollo Forestal y Manejo de las Areas Protegidas (INDEFOR-AP), Bata, Equatorial Guinea; 4 African Aquatic Conservation Fund, Chillmark, Massachusetts, United States of America; 5 Centre for Ecology and Conservation, Faculty of Environment, Sustainability and Economy, University of Exeter, Penryn Campus, Cornwall, United Kingdom; 6 Bioko Biodiversity Protection Program, Malabo, Bioko Norte, Equatorial Guinea; 7 Department of Biodiversity, Earth and Environmental Science, Drexel University, Philadelphia, Pennsylvania, United States of America; 8 Universidad Nacional de Guinea Ecuatorial, Malabo, Equatorial Guinea; 9 US National Fish and Wildlife Service, Division of International Conservation, Falls Church, Virginia, United States of America; 10 Ocean Ecology Network, Research Affiliate of NOAA-National Marine Fisheries Service, Southwest Fisheries Science Center, La Jolla, California, United States of America; MARE – Marine and Environmental Sciences Centre, PORTUGAL

## Abstract

Bioko Island (Equatorial Guinea) hosts important nesting habitat for leatherback sea turtles, with the main nesting beaches found on the island’s southern end. Nest monitoring and protection have been ongoing for more than two decades, although distribution and habitat range at sea remains to be determined. This study uses satellite telemetry to describe the movements of female leatherback turtles (n = 10) during and following the breeding season, tracking them to presumed offshore foraging habitats in the south Atlantic Ocean. Leatherback turtles spent 100% of their time during the breeding period within the Exclusive Economic Zone (EEZ) of Equatorial Guinea, with a core distribution focused on the south of Bioko Island extending up to 10 km from the coast. During this period, turtles spent less than 10% of time within the existing protected area. Extending the border of this area by 3 km offshore would lead to a greater than threefold increase in coverage of turtle distribution (29.8 ± 19.0% of time), while an expansion to 15 km offshore would provide spatial coverage for more than 50% of tracking time. Post-nesting movements traversed the territorial waters of Sao Tome and Principe (6.4%of tracking time), Brazil (0.85%), Ascension (1.8%), and Saint Helena (0.75%). The majority (70%) of tracking time was spent in areas beyond national jurisdiction (i.e. the High Seas). This study reveals that conservation benefits could be achieved by expanding existing protected areas stretching from the Bioko coastal zone, and suggests shared migratory routes and foraging space between the Bioko population and other leatherback turtle rookeries in this region.

## Introduction

The leatherback sea turtle (*Dermochelys coriacea*) is a globally distributed, long-distance migratory pelagic species [1–8], which is of conservation concern [9] and is identified as Vulnerable to extinction by the IUCN [10]. Leatherback turtle nesting behaviour and ecology across much of the world has been described, as have the location of critical beach habitats and trends in nesting numbers for numerous populations [11–16]. These life phases, however, represent a limited proportion of a turtle’s entire life. In contrast, descriptions of leatherback turtle behaviour and ecology at sea were limited until the advent of satellite tracking technologies. Fastloc GPS and satellite-linked positioning tags have revealed the migrations of the species between feeding and nesting grounds [3, 5–7,17–20], depth and temperature time series have been used to describe adaptive diving behaviour associated with tracking of foraging opportunities [5, 21–23], and coupling of location and dive data with distribution data of human activities has revealed hotspots of conservation concern [1, 2, 20, 24, 25].

Human exploitation, directed or accidental, has resulted in population declines of leatherback turtles [9, 15, 16]. These declines have been severe among populations in the Pacific Ocean, which suffered reductions in nest numbers of up to 78% between 1984 and 2011 [9], and do not demonstrate signs of recovery despite continued conservation efforts [26]. Although many rookeries in the Atlantic ocean remained stable or showed signs of increase [11, 12, 27, 28], recent declines have also been observed for some populations in this basin [29]. Nonetheless, the majority of leatherback turtles are now found in the Atlantic Ocean [19, 27], and the world’s highest concentration of leatherback nesting activity occurs in the Gulf of Guinea [30], representing approximately 60% of all nesting females in this basin. Within the region, Gabon’s population is the largest (estimated at 36,185–126,480 clutches per year [30]) and has been studied extensively [19, 27, 30, 31], but its connectivity and overlap with other potentially large rookeries in the region, like Bioko Island in Equatorial Guinea [32] and Republic of the Congo [33], are yet to be established.

Equatorial Guinea’s Bioko Island hosts important nesting habitat for four species of sea turtle [32, 34], including the leatherback turtle. Although few estimates of population size are currently available, up to 444 female leatherback turtles have been observed nesting on the island each year [32] making this the second most important rookery for the species in the region (after Gabon), and suggesting that this population may be of regional and global importance [34]. Land-based leatherback turtle nest monitoring and protection of nesting females have been carried out on the island for more than two decades [32, 34] by the conservation groups Bioko Biodiversity Protection Program (BBPP) and Amigos de Doñana, although the number of leatherback turtles nesting on the island declined annually from 2008–2014, likely due to accidental and directed capture in artisanal and commercial fisheries [32].

The most important nesting beaches, located along the southern coast [32, 34], are found within the Caldera de Luba Scientific Reserve, managed by the Institute for Forestry Development and Protected Area Management (INDEFOR-AP), under the Ministry of Agriculture, Husbandry, Forestry and Environment. The reserve is mostly land-based, but a small marine component (120 Km^2^, 20% of the reserve) covers coastal waters to the south of the island, up to 2km offshore. Recently, proposals have been put forward to expand the marine portion of the reserve up to 5km offshore [35], though enforcement of protection remains a major issue for the park.

As such, marine protection is lagging behind terrestrial efforts, despite evidence that, upon leaving the nesting beach, sea turtles are subject to intentional and incidental fisheries bycatch [19, 20], as well as impacts from pollution, shipping and the oil industry [36–44]. Telemetry data collected from leatherback turtles nesting in Gabon has shown that inter-nesting [31] and post-nesting migrations [19, 20], as well as nest-site fidelity [31], follow complex patterns of coastal and open-ocean habitat use that require bespoke conservation planning and suggest that other populations may similarly benefit from regional management plans. While the Gabonese and Equatoguinean leatherback populations occupy geographically similar habitats, at least during their nesting periods, inter-population links have not yet been observed. Consequently, the intra- and post-nesting movements of the Equatoguinean population and the threats it may face in national and international waters also remain unclear. A more detailed understanding of temporal and spatial use of Equatorial Guinea’s territorial waters is essential to develop effective protection and management strategies for its endangered leatherback turtle population, particularly with respect to conservation planning in coastal areas.

Here, we address the information gap for the Equatoguinean leatherback population by deploying satellite tracking tags on females nesting in Bioko Island. We use tracking data to characterise the distribution and extent of movement grounds for female leatherbacks during the nesting season, as well as to describe their movement patterns during their migratory phase. We further use these data to determine the effectiveness of the existing Nature Reserve in protecting nesting grounds for the species and evaluate the benefits of potential expansion plans, as well as attempt to characterise the risk posed to the population by fishing activities during their migration.

## Methods

### Ethics statement

Attachment of satellite transmitters to leatherback turtles has undergone ethical review by the Ethical Review Committee of the College of Life and Environmental Sciences at the University of Exeter. All fieldwork was subject to full risk assessment according to the Management of Health and Safety at Work Regulations (1999) United Kingdom.

### Study area and tagging procedure

**Fig 1:**
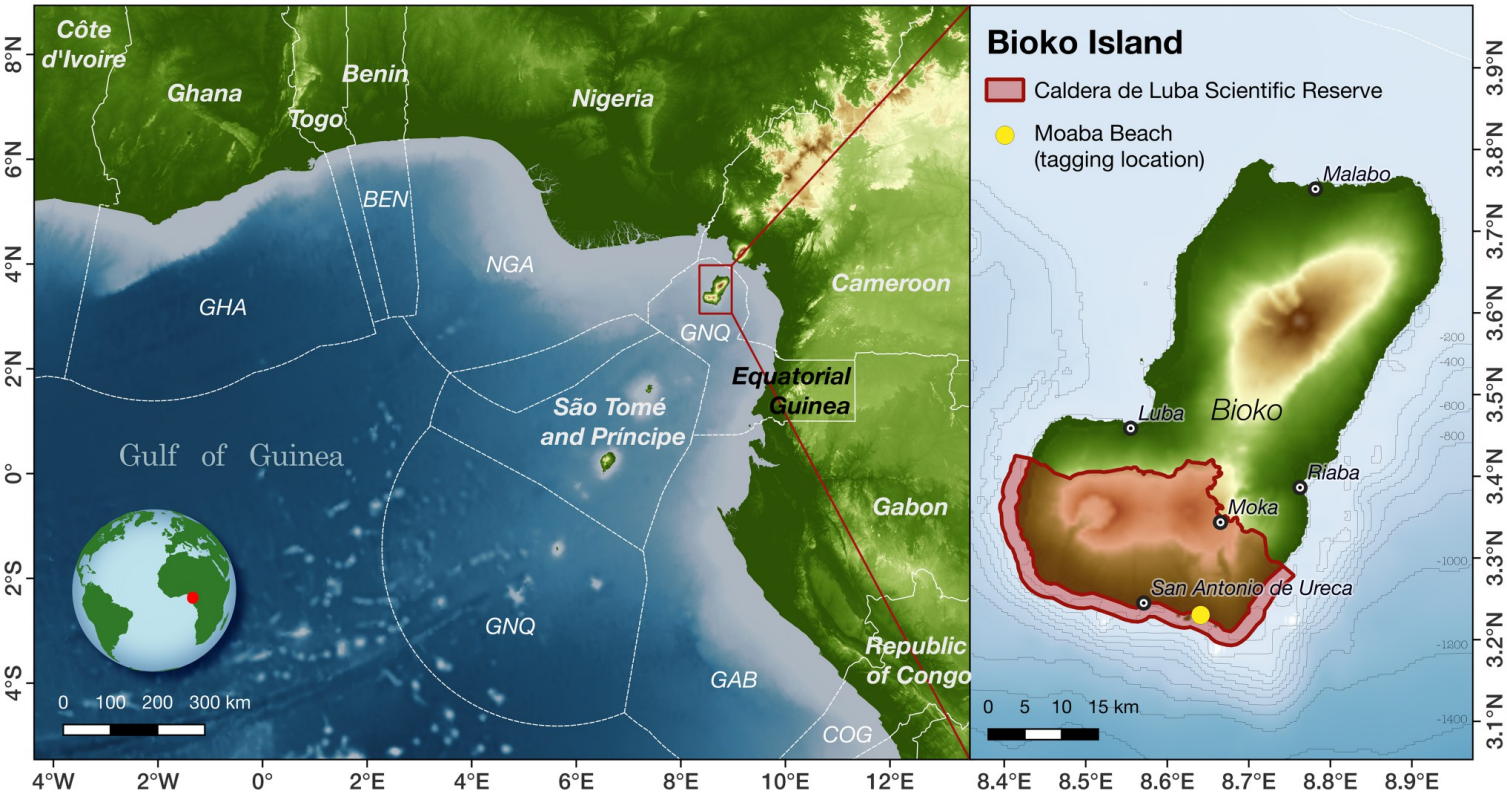
Tagging location of female leatherback turtles (*Dermochelys coriacea*) from Bioko Island, Equatorial Guinea. Moaba beach is indicated by yellow dot on the right map panel. Left panel shows the location of Bioko Island within the Gulf of Guinea. GHA = Ghana, BEN = Benin, NGA = Nigeria, GNQ = Equatorial Guinea, GAB = Gabon, COG = Republic of Congo. Basemap created in QGIS using freely available data from Natural Earth (www.naturalearthdata.com) and the General Bathymetric Chart of the Ocean (www.gebco.net).

Intensive night-time beach patrols for nesting leatherback turtles took place at Moaba Beach, Bioko Island, which is a 3-km long nesting beach in the Caldera de Luba Scientific Reserve, (Fig 1). Whenever a turtle was observed crawling on the beach or nesting, patrols were paused to allow the turtle to nest. Once egg laying had finished, nesting females were fitted with satellite tags using direct carapacial attachment [19], whereby tags were secured by nylon loops passed through holes drilled in the central dorsal ridge of the turtle’s carapace [45]. Morphometric data (e.g. curved carapace length) were also collected from each tagged turtle. Ten nesting females were equipped with Argos satellite tags (SPOT 6; Wildlife Computers, WA, USA) in January 2019, during the peak of the nesting season. Satellite tags transmitted messages to overpassing Argos-enabled satellites whenever the animal was at the sea surface or on land. Argos-derived locations have varying spatial error, depending primarily on the number of messages received, which is broadly a function of the length of time a tag is exposed to the air while relevant satellites are within detection distance. Location error for each position is encoded into 6 location classes (Z, 0, B, A, 1, 2, 3, in increasing order of accuracy), and ranges on average from over 14 km (LC Z—B) to 400 m (LC 3) [46]. The Argos system estimates the location of tagged animals based on doppler shift of message frequencies, and applies Kalman smoothers to improve accuracy of estimations [47]. Tags were also programmed to record and transmit information on the percentage of time the tag was dry (i.e. aerially exposed either at the sea surface or on land). Tags were set to transmit data without limit to the number of daily upload attempts and with no duty cycling.

### Data processing and filtering

Argos-derived locations were filtered to eliminate positions with high or unquantifiable location accuracy (Argos LC 0 and Z), positions on land during the inter-nesting period, and positions resulting in unrealistic movement speeds (>15m/s). Locations were regularised using the bsam state-space-modelling package in R [48, 49]. Locations from bsam were generated at 4 hours intervals using a hierarchical, first difference correlated random walk model with 1000 samples for adaptation and burn-in to limit the effects of initial conditions, 5000 generated samples after adaptation with thinning factor of 5 to reduce autocorrelation effects, and smoothing parameter of 0.2 [sensu 49].

Behavioural-switching models were applied to the data to distinguish between nesting and migratory movements [50], but distinctions between the two phases were not clear in the estimates due to the highly mobile inter-nesting phases displayed by the species. Hence, net squared displacement (NSD, i.e. summative between-points straight-line minimum distance from the tag attachment location) was used to estimate the beginning of migratory movements [51–54]. NSD was calculated for each location for all individual turtles, and the track was segmented based on patterns in mean and variance of NSD through the Lavielle method [55, 56].

Lavielle’s method allows non-parametric segmentation of a time series using the penalized contrast method [57, 58]. This method finds the best segmentation of a time series of focus variables (e.g. residence time or step length) into K segments. It searches the segmentation for which a contrast function (measuring the contrast between the actual series and the model underlying the segmented series) is minimized. Lavielle segmentation was conducted though the adehabitatLT package [59], setting a minimum step length of 12 hours and a maximum of 6 possible segments (this number was selected to allow for potential multiple switches between foraging areas after migration). Visual inspection confirmed that for all turtles tracked for a long enough period, the first breakpoint was always coincident with the beginning of movements away from the nesting grounds, indicating the beginning of migratory movements. These times were used to divide tracking datasets into inter- and post-nesting tracks.

### Inter-nesting ecology

To describe turtle movements between nests, nesting attempts recorded in the track were identified using timelines of hourly percentage dry time. Leatherback turtle nesting events take between 72 and 187 minutes [60, 61], as such, nesting events were identified by peaks in % dry time higher than 60%. This threshold assumes a hypothetical nesting event spread between two consecutive hours, with at least 36 minutes of dry time in any hour. Once the timing of nesting attempts was determined, tracking locations within 2-hour intervals centred on nesting times were extracted from the unfiltered Argos location dataset to confirm turtles were on land at the time.

In the case that two or more nesting attempts were identified within 10 days of each other (i.e. the known interesting period for leatherback turtles in Gabon [41]), only the later event was considered a likely successful nesting, while the earlier event(s) were classified as failed attempts. Similarly, events for which % dry time exceeded 60% but equalled 0% in the hour prior and following were also considered to be likely failed nesting attempts. Individual tracking sets were then cut at each estimated nesting time to investigate movement patterns between consecutive attempts. Two datasets were used to estimate turtle movements between nests and the timing of nesting events: (i) SSM-derived locations and (ii) tag-measured % dry time data time series; these were not temporally aligned (SSM locations sampled at 4h intervals, dry/wet timelines summarised daily) and not directly linked spatially. Therefore, minor inconsistencies arose between timing of locations and nest events, though they remained closely aligned.

Home range and core area distributions were generated for each tagged turtle by applying continuous time-movement models (CTMM; [62]) in R to filtered Argos location data. A variety of models in CTMM were fitted (see [62]) to individual tracking datasets and were parameterized to account for location error according to Argos class along with knowledge of the cyclical movements undertaken by leatherback turtles as they move towards and away from the beach on each subsequent nesting event using a mean inter-nesting interval of 11.5 days, similar to what observed for the Gabonese rookery (9–11 days) [41]. This interval was adjusted for some individuals to allow for better fits of the model to data (S1 Table). The most suitable model was selected as one having the lowest AIC score. In cases where multiple models had similar AIC scores (ΔAIC<4), the most optimal model was selected by visually inspecting their fit to variograms. Core areas were defined as the contour equivalent to the 50% utilisation distribution (UD), while home range was defined at the 95% UD contour.

### Post-nesting ecology

Post-nesting datasets (i.e. SSM regularised locations dated after the inferred migration start date) were created for each individual, and displacement from the tag attachment location was calculated for each location. Displacement trajectories were plotted and inspected to identify potential commonalities in dispersal strategy among individuals. Dispersal movements were examined in QGIS and the percentage of locations situated within the Exclusive Economic Zones (EEZ) of relevant countries in the Central Atlantic Ocean was calculated using simple point-in-polygon principles.

### MPA cover and fishing threat

Spatially referenced shapefiles of the Caldera de Luba Scientific Reserve were used to quantify the overlap between turtle movements and core areas, and the existing protected area. Given leatherback turtle locations were regularized to 4-hour intervals through BSAM, the number of locations identified inside the boundaries of the reserve was used as a direct measurement of the time spent within the reserve.

Potentially critical areas for protection of leatherback turtles were estimated by calculating the union and the intersection of the individual core areas obtained from kernel density calculations resulting from CCTM analysis. The union of these areas represented the total extent of core habitat for the species in proximity of the island, while the intersection of core areas was taken to represent the minimal candidate core area for protection.

To explore the potential benefits of expansions to the marine component of the reserve, additional polygons were created by buffering the sea-facing boundary of the reserve at intervals of 1 km from 2 km (the limit of the current protected area) to 10 km from land, and at 15 km and 20 km. The number of leatherback turtle 4-hour regularised locations within these boundaries (i.e. amount of time spent in them), as well as the portion of union and intersection of core areas covered by them were calculated. The calculations were also repeated for the current expansion proposal outlined in the latest marine atlas for spatial planning of Equatorial Guinea’s waters [35].

The overlap between active fishing areas and estimated turtle locations was investigated to estimate the potential interaction between fishing activities and leatherback turtles during their migration. Locations were estimated at 1-day intervals using BSAM (see above for details) and overlaid on long-term mean fishing effort maps for the region created from data produced by Global Fishing Watch (Copyright [2022], Global Fishing Watch, Inc., www.globalfishingwatch.org) [63]. Net square displacement curves were computed and visually assessed to infer switches in turtle behaviour from directed movement away from nesting grounds (characterised by strong positive relation between displacement and time) to slower, non constant patterns of displacement that may indicate area-restricted movements [53].

Monthly fishing effort (in hours at 0.1-degree resolution) for longline and purse seine fishing was obtained from the Global Fishing Watch for the observed migration period (February—August) between 2018–2022. Fishing data resolution was then adjusted to 0.5 degrees to encompass the average confidence interval of position estimates obtained from BSAM and cumulative hours of fishing effort were computed for each new cell. Subsequently, yearly summaries of cumulative fishing effort were calculated as the sum of monthly effort in each year, and long-term averages were calculated as the mean fishing effort per cell for longliners, purse seiners, and both methods combined.

Overlap between turtle tracks and fishing effort was visually estimated to identify notable features.

## Results

### Summary of tagging

Ten female leatherback turtles (mean ± sd; CCL 145.3 ± 7.04 cm) were tagged in January 2019 at Moaba Beach, Bioko Island (Equatorial Guinea). Tags transmitted for 132 ± 50 days (mean ± sd; range 20–205; Table 1), yielding 1,103 ± 526 locations per individual (range 131–2,016). After filtering, 822 ± 336 locations were available for each individual (range 109–1,302, 8,220 in total). Both inter-nesting and post-nesting behaviour was observed in 8 turtles. One turtle began migratory movements immediately after tagging (turtle 8), and one tag stopped transmitting before the turtle had left the offshore area adjacent to the nesting area (turtle 1).

**Table 1:** Satellite tagging of leatherback turtles (*Dermochelys coriacea*) in Bioko—Equatorial Guinea.

Deployment Duration ^f^	(days)	20	98	163	122	140	205	158	169	135	112
Total Distance ^e^	(km)	899	4,302	9,751	6,050	8,942	7,658	8,669	6,320	8,028	7,292
Final Displacement ^d^	(km)	63	2,238	2,087	1,758	3,096	2,447	2,451	3,326	5,137	3,303
Observed Migration Duration	(days) ^c^	0	74	132	108	114	171	142	169	104	100
Last Location Date		04/02/2019	20/04/2019	25/06/2019	16/05/2019	02/06/2019	10/08/2019	23/06/2019	01/07/2019	29/05/2019	08/05/2019
Migration Group ^b^		NA	Central Atlantic	Central Atlantic	Central Atlantic	Central Atlantic	Central Atlantic	Central Atlantic	South America	South America	South America
Migration Start Date		NA	05/02/2019	12/02/2019	28/01/2019	09/02/2019	20/02/2019	02/02/2019	13/01/2019	15/02/2019	30/01/2019
Observed Nesting Duration ^a^	(days)	20	24	30	14	25	34	16	0	32	14
Estimated Nesting Attempts		2	4	6	5	5	4	4	1	4	3
Tag Date		15/01/2019	12/01/2019	13/01/2019	14/01/2019	14/01/2019	16/01/2019	16/01/2019	13/01/2019	13/01/2019	15/01/2019
CCW	(cm)	106	105	104	103	105	101	103	104	118	110
CCL	(cm)	154	144	143	139	142	137	140	145	158	152
PTT		84274	84268	84269	84272	84273	101203	101207	84270	84271	101201
ID		1	2	3	4	5	6	7	8	9	10

Summary statistics for each turtle tagged between Jan 12^th^ and Jan 16^th^ 2019, including morphometric measurements.

PTT, Platform Transmitting Terminal; CCL, Curved Carapace Length; CCW, Curved Carapace Width

^a^ Time interval between tag attachment and commencement of post-nesting migration

^b^ * Turtles are grouped based on the migratory strategy followed upon leaving nesting grounds, either remaining in the Central Atlantic Ocean, or heading towards likely foraging grounds near South America.

^c^ Time interval between estimated commencement of post-nesting migration and end of tracking data

^d^ Minimum straight-line distance between tagging location and last location received

^e^ Along track sum of minimum straight-line distance

^f^ Time interval between tag deployment and last transmission received form tag

Leatherback turtles spent 100% of their time during the breeding period inside Equatorial Guinea’s EEZ. During post-nesting migratory movements, leatherback turtles occupied the territorial waters of Sao Tome and Principe (6.4% of locations), Ascension Island (UK; 1.8%), Brazil (0.85%), and Saint Helena (UK; 0.75%), although they were found in international waters (outside of any country’s EEZ) for the majority of the time (70%).

### Inter-nesting movements and use of protected areas

Leatherback turtles remained within their offshore breeding area for 20.9 ± 10.3 (mean ± sd) days after tag attachment (range 0–34 days), before commencing post-nesting migration. Within this period, leatherback turtles laid 1–4 clutches (2.54 ± 1.1 nests mean ± sd) at intervals of 11.5 ± 1.8 days (Table 1).

**Fig 2:**
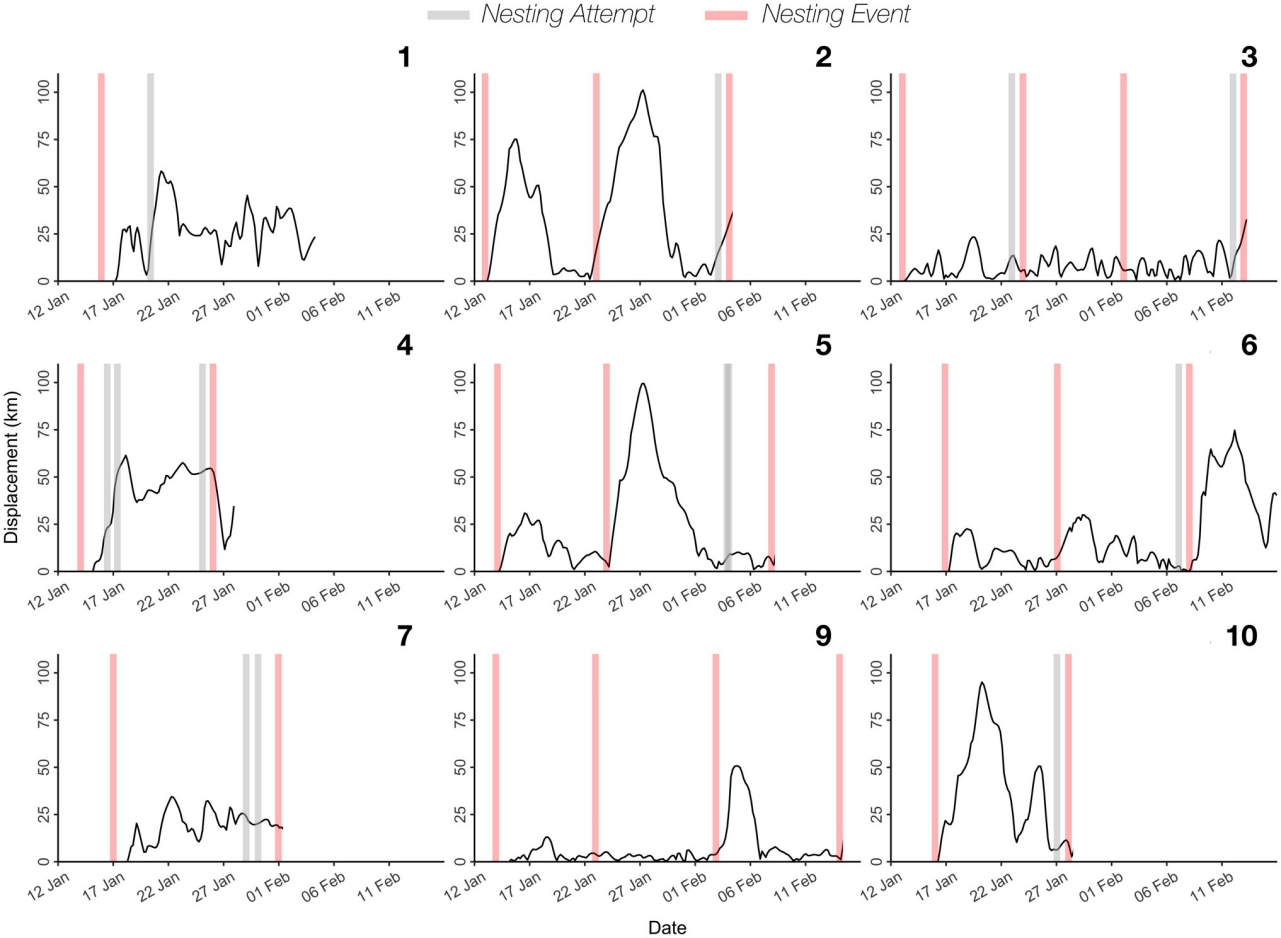
Inter-nesting displacement and nesting events of leatherback turtles (*Dermochelys coriacea*) from Bioko Island, Equatorial Guinea. Displacement was calculated as the minimum straight-line distance (km) from the tagging location. Grey and red shaded vertical bars indicate putative nesting attempts and assumed successful nesting events, respectively. Displacement series in the figure were terminated before the beginning of migratory movements away from the breeding area. Order as in Table 1.

Displacement curves revealed differences in behaviour between nesting attempts, with turtles moving both offshore and/or remaining near shore (Fig 2). Turtles undertook offshore trips up to 108 km away from their original tagging locations between consecutive nesting attempts (mean ± sd; 66.3 ± 27.0 km), and displayed relatively high levels of horizontal movement while in the breeding area, covering a minimum distance of 482 ± 222 km (mean minimum distance ± sd) on average, or 25.37 ± 6.9 km day^-1^.

**Fig 3:**
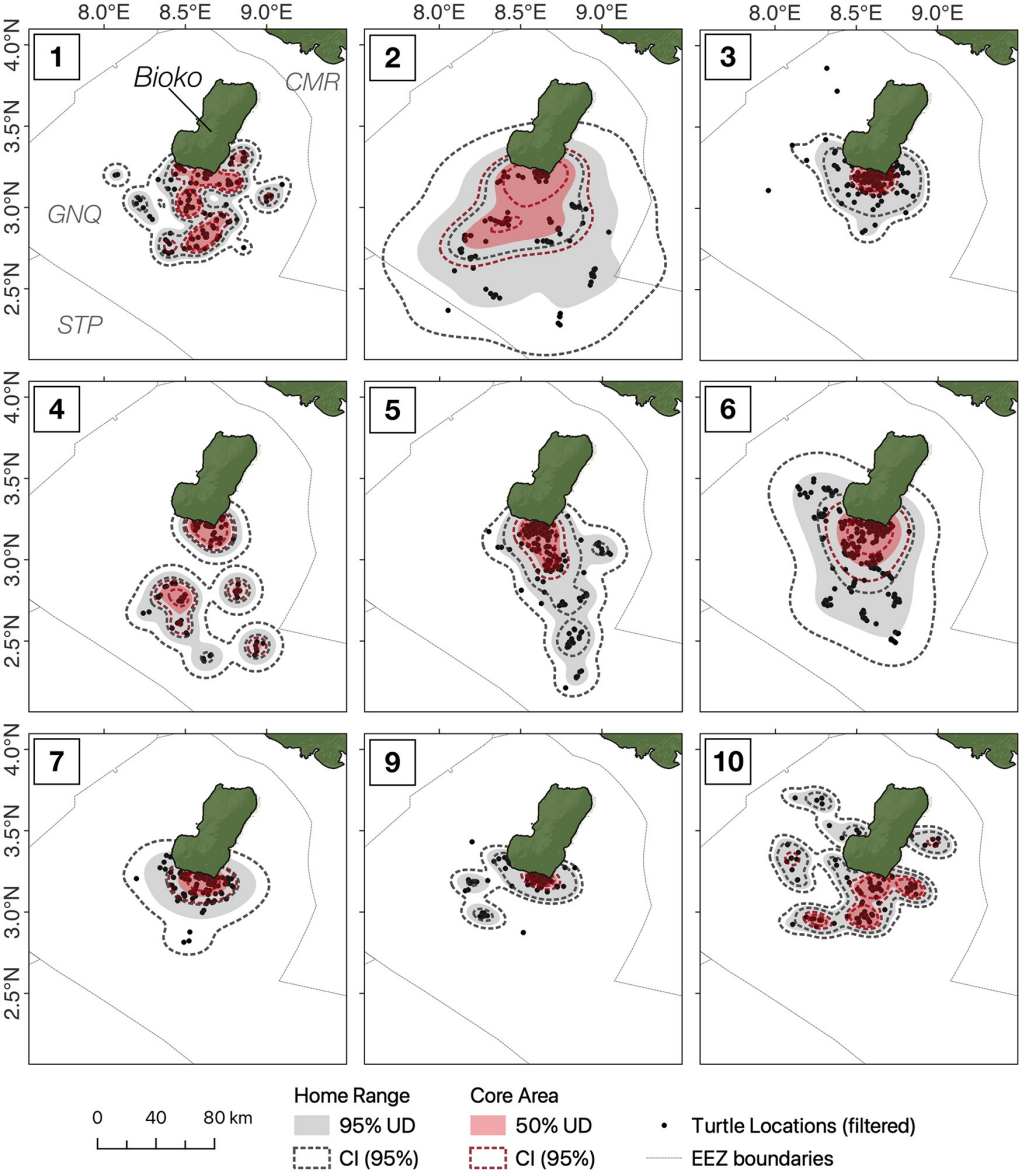
Utilisation distribution contours of female leatherback turtles (*Dermochelys coriacea*) during their reproductive season in Bioko Island, Equatorial Guinea. Filtered turtle locations obtained from satellite tracking are indicated by black dots. Core areas and home ranges of individuals obtained from ctmm modelling are displayed in red and grey polygons, respectively. Turtle 8 is not included as the individual left the nesting grounds less than 24 hours following tagging. GNQ = Equatorial Guinea, STP = São Tomé and Príncipe, CMR = Cameroon. Basemap created in QGIS using freely available data from Natural Earth (www.naturalearthdata.com) and the General Bathymetric Chart of the Ocean (www.gebco.net).

Core areas of distribution (50% UD) for all tagged turtles were focused on the south-east of the island, in proximity of the nesting beach where they were tagged and extending 10 km offshore (Fig 3), although areas further offshore (up to 75 km from the coast) were also identified in this category for some individuals (e.g. ID: 4, 5, Fig 3). Overall, the core area of utilisation for female leatherback turtles within nesting grounds covered 3,888 km^2^, while their home range spanned 15,236 km^2^ (Fig 3). Of the 9 turtles successfully tracked into their migration, all but 1 appeared to commence migratory movements immediately after their final nesting attempt (Fig 2).

**Fig 4:**
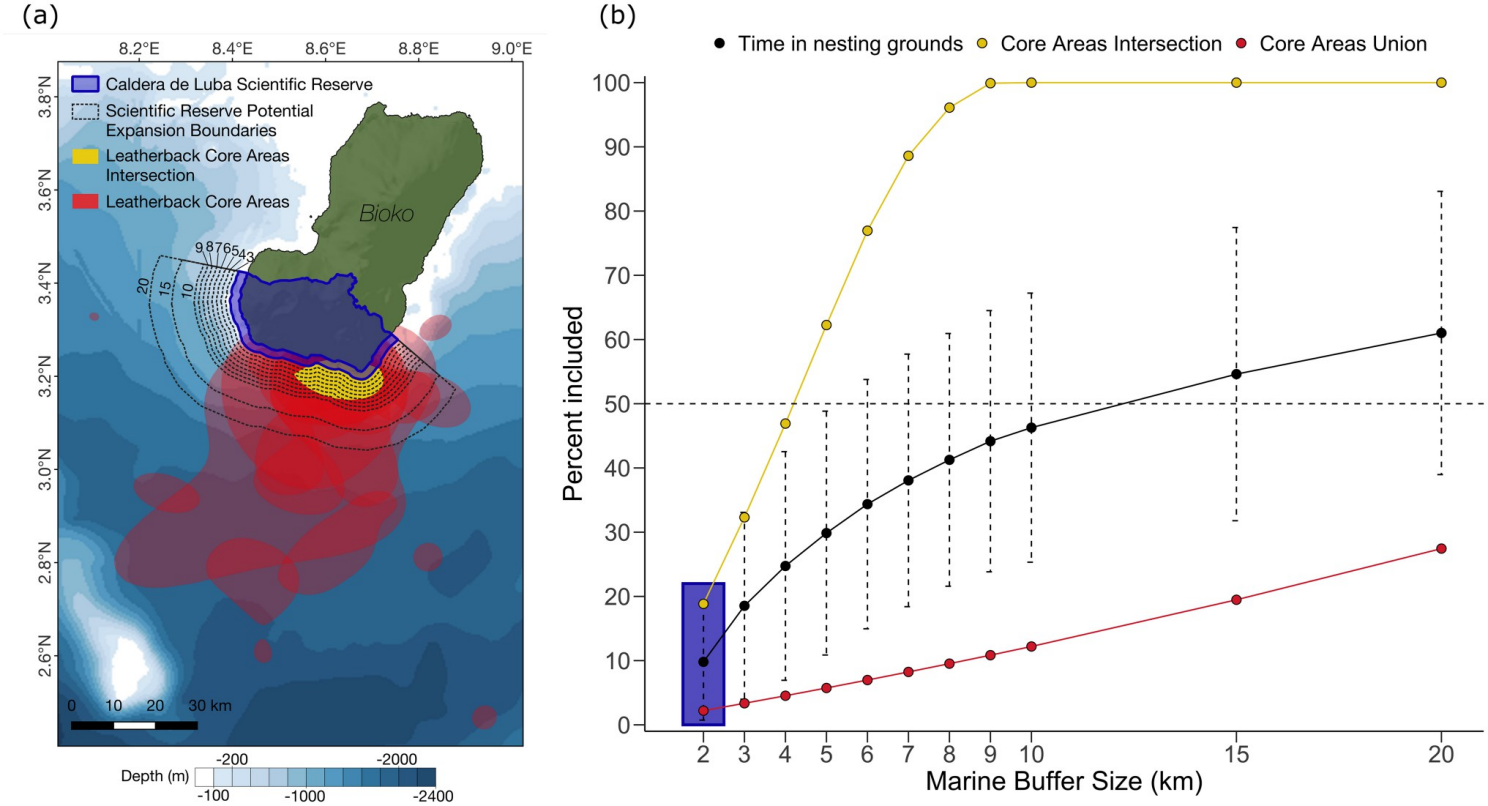
Protection offered by existing and proposed marine boundaries of the Caldera de Luba Scientific Reserve to nesting leatherback turtles (*Dermochelys coriacea*) in Bioko Island, Equatorial Guinea. a) The current boundaries of the Caldera de Luba Scientific Reserve are indicated in blue, while proposed potential expansions of its marine component are represented by black dotted lines placed at 1km intervals from the current boundary up to 10 km from the shore, and also at 15 km and 20 km from the shore. Red polygons represent the core utilisation areas for all tagged individuals, yellow polygon indicates the intersection of all core utilisation areas. b) Protection offered to turtles by increasing marine buffer radii for the Caldera de Luba Scientific Reserve, quantified as the average percentage of time tracked turtles spent within them (black dots, error bars represent SD), percentage of the intersection of core areas covered by the buffer (yellow dots), and percentage of the union of core areas covered by the buffer (red dots). The buffer size currently in existence (i.e. 2 km buffer from shore) is highlighted in the blue rectangle. Basemap created in QGIS using freely available data from Natural Earth (www.naturalearthdata.com) and the General Bathymetric Chart of the Ocean (www.gebco.net).

Leatherback turtles were found within the marine area of the Caldera de Luba Scientific Reserve for 9.8 ± 9.1% (1.6–31.2%) of their tracking time, and the reserve covered just 18.8% of the core breeding grounds (identified as the intersection of core areas for each individual, estimated through ctmm). All scenarios explored for the expansion of the existing reserve (which currently extends 2 km from the coast) increased the spatial and temporal coverage with turtle distribution (Fig 4a). Moving the border of the reserve to 5 km offshore led to a more than threefold increase in coverage of turtle distribution (29.8 ± 19.0% of time), while an expansion to 15 km provided spatial coverage for more than half the tracking time (mean ± sd; 54.6 ± 22.8%). An expansion to 10 km would be needed to cover the entire intersection of core areas for all tracked individuals (Fig 4a), in which turtles are estimated to spend 35.0% of their time (sd = 18.1, range = 14.0–74.4%). If all individual core areas were to be spatially congruent with an expanded MPA (red polygon, Fig 4a) then protection would be afforded for leatherback turtles for 80.4 ± 15.1% of the time spent in breeding areas. The current expansion proposal was found to cover 63.7% of the core nesting grounds and 6.4% of the union of core areas, accounting for 31.4% of tracking time.

### Post-nesting movements and fishing threat

**Fig 5:**
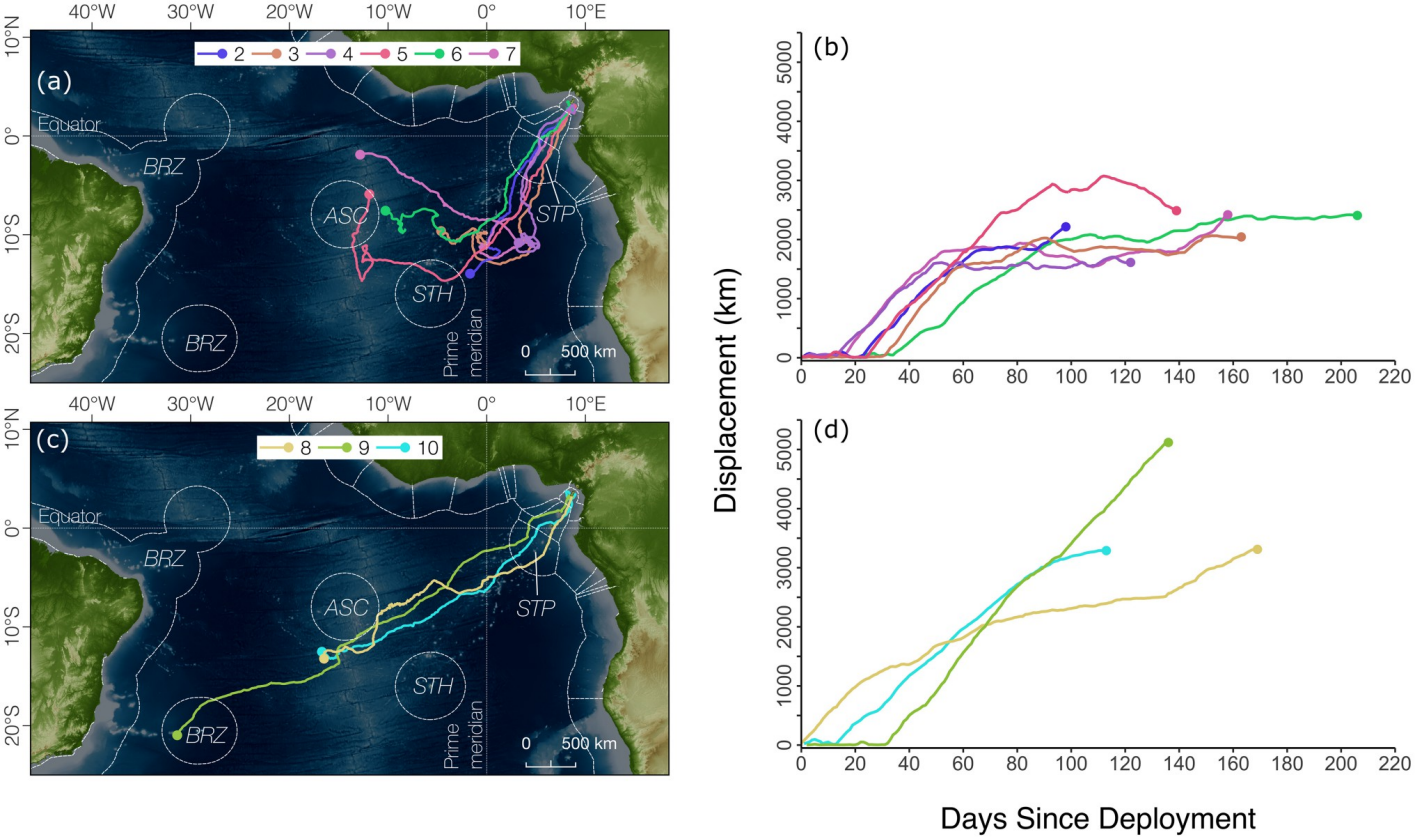
Post-nesting migratory strategies of leatherback turtles (*Dermochelys coriacea*) satellite tagged in Bioko, Equatorial Guinea. (a, c) Satellite-tracked movements and (b, d) daily displacement from the time turtles left their breeding grounds showing 2 dispersal groups: (a, b) offshore waters in equatorial Atlantic, and (b, c) direct dispersal towards South America. Colour codes for individuals are conserved among panels. BRZ = Brazil, ASC = Ascension, STH = St. Helena, STP = São Tomé and Príncipe. Basemap created in QGIS using freely available data from Natural Earth (www.naturalearthdata.com) and the General Bathymetric Chart of the Ocean (www.gebco.net).

For all turtles tracked into their migration, an initial burst of directed movement away from Bioko Island towards the central Atlantic Ocean could be detected from displacement curves between February and early April (Fig 5), followed by slower rates of displacement that suggest potential feeding activity for all but one turtle.

Two groups of post-nesting dispersal behaviours could be distinguished among tracked individuals. The first group (n = 6) was observed to spend considerable time in habitats within the Central Atlantic Ocean (Fig 5a), and displayed more tortuous movements that were constrained to within 2316 ± 457 km of their tagging locations (maximum displacement range 1704–3075 km). The second group (n = 3) was characterised by directed movement across the Atlantic towards habitat off the coast of South America, dispersing up to 5119 km from their tagging site (minimum 3291 km, mean 3907 ± 1050; Fig 5).

**Fig 6:**
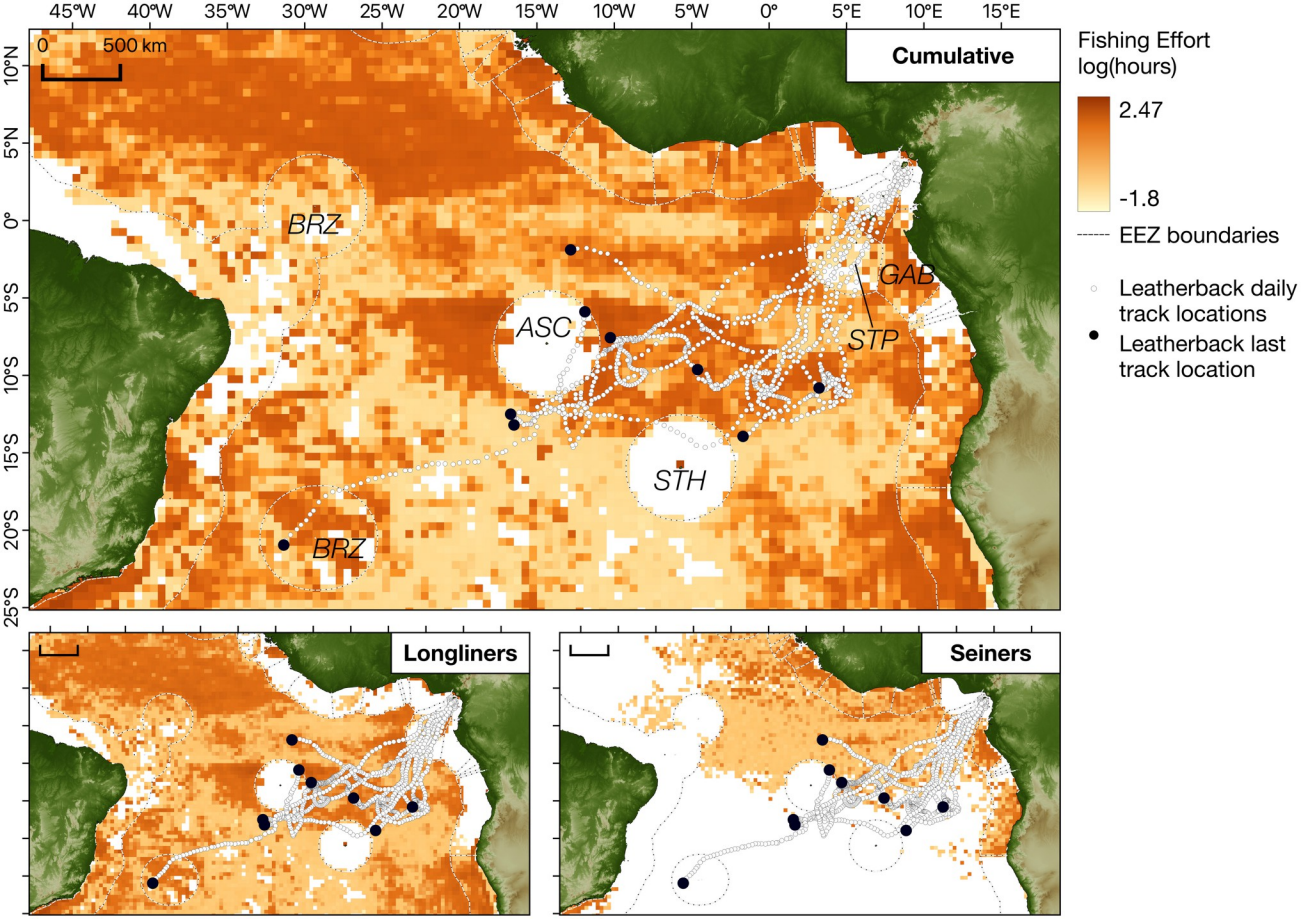
Overlap between leatherback turtle (*Dermochelys coriacea*) tracked from their nesting grounds in Bioko Island and longline and seine fisheries in the Central Atlantic Ocean. Cumulative fishing effort (top panel) for longline and seine fishing vessels for the tracking period aggregated and shown in 0.5x0.5° cells (bottom-left and bottom-right show effort separately for longline and seine, respectively). Please note fishing effort is depicted on a logarithmic scale. Original fishing dataset from Global Fishing Watch www.globalfishingwatch.org. Black dashed lines represent limits of Exclusive Economic Zones (EEZ) of the bordering countries. Regularised daily locations of tracked turtles are indicated as white dots. BRZ = Brazil, ASC = Ascension, STH = St. Helena, STP = São Tomé and Príncipe, GAB = Gabon. Basemap created in QGIS using freely available data from Natural Earth (www.naturalearthdata.com) and the General Bathymetric Chart of the Ocean (www.gebco.net).

Most fishing activities appeared to be focused on international waters near Brazil and between the EEZs of Ascension Island (UK) and Saint Helena (UK) (Fig 6). As a result, overlap of turtle movements with fisheries may be high, especially near the end of the recorded tracks, which could represent important foraging areas for the turtles (Fig 6). Longline and purse seine fishing effort were not equally distributed, with purse seiners targeting EEZ waters along west Africa much more than the high seas, and longlining operations being particularly active in the high seas near Ascension Island and Saint Helena, and off the coast of Brazil (Fig 6, bottom-right and bottom-left, respectively).

## Discussion

### Nesting ecology and in-country conservation planning

Leatherback turtles commonly occupy large areas during their inter-nesting period, sometimes covering distances greater than 200 km and often crossing exclusive economic zone boundaries into waters of neighbouring countries [24]. Nesting females have been observed to routinely cross from Gabon into Congo [31], French Guyana into Suriname [24], and from Grenada into at least seven other nearby countries [24]. This study, however, revealed that leatherback turtles in Equatorial Guinea, while still undertaking long movements during inter-nesting periods, remained exclusively within national waters. Leatherback turtles in the country furthermore seem to have relatively restricted areas of occupancy within the EEZ. Female turtles’ core occupancy areas are highly concentrated on the south-east of Bioko Island, and their home ranges rarely reach beyond EEZ boundaries into neighbouring countries. These results largely agree with the species distribution models previously created for leatherback turtles in the country [43]. However, these predictive models underestimate the importance of near-shore habitats in proximity of Bioko Island, thereby reinforcing the need to collect locally relevant data to underpin more effective conservation strategies. Together, restricted distribution and circumscription to a single EEZ represent a notable advantage regarding the protection of the population and its nesting grounds, as significant and efficient protection measures can be enacted by the country acting independently, without the need for additional international cooperation.

During their nesting period in the country’s EEZ, leatherback turtles may suffer from directed and accidental catch in artisanal coastal fisheries, as well as incidental boat strikes. Though both sectors are not currently highly active in the south of Bioko Island [35], where turtles likely spend most of their time, active turtle poaching still occurs in the area. Oil and gas operations are also largely absent form this area [35], suggesting potential for expansion of spatial protection that may not result in significant loss to other stakeholders. The waters south of Bioko also host other marine megafauna and reef systems of conservation interest [35] that too would benefit from greater spatial protection. While nesting beaches on Bioko Island are already under the protection of the Caldera de Luba Scientific Reserve, leatherback turtles are not currently offered spatial protection at sea within Equatorial Guinea’s EEZ. Here they may be vulnerable to accidental and directed fishing, as well as negative impacts form gas and oil extraction activities [32, 34]. The Scientific Reserve includes a small proposed marine portion in the form of a buffer, extending 2 km into coastal waters along its sea-facing boundary, but this zone was found to likely only overlap a small portion of leatherback space use. Suggestions for the expansion of the marine component of the scientific reserve have recently been proposed, which are believed to offer greater protection to leatherback turtles and other marine megafauna found in the country [35]. The findings of this study suggest that the MPA expansion scenario proposed in the latest marine atlas for spatial planning of Equatorial Guinea’s waters [35] has the potential to significantly increase the spatial protection offered to nesting females, covering the vast majority of their core occupancy area. The proposals for expansion of the reserve were found to be largely comparable to the 5km buffer expansion examined here, including a similar proportion of leatherback use areas (63.7 vs 62.3 and 6.4 vs 5.7 for the intersection and union of individual core areas, respectively) and individual tracking time (31.4 ± 18.6 vs 29.9 ± 19.0). While this expansion would represent a substantial increase in spatial protection for nesting females, the simulations presented in this study suggest that further expansions to 10–15 km should also be considered if management aims to maximise the spatial protection offered to leatherback turtles and other coastal species. This expansion would comprise 50–58% of tracking time for the individuals followed in this study and fully encompass the core utilisation area of the species.

Expansion of the Caldera de Luba Scientific Reserve should however be also supported by financial resources that can be used to enforce protection within its boundaries. While a management plan for the reserve was ratified in 2021, patrolling of the reserve and enforcement of protection has been lacking due to lack of resources, particularly at sea.

### Migratory movements and overlap with fisheries

While leatherback turtles in the Pacific Ocean display consistent population-specific migratory routes and share feeding grounds [7], populations in the Atlantic Ocean are instead characterised by high levels of intra-population diversity in post-nesting movement [18]. For example, satellite tracking of Gabonese female leatherbacks revealed the existence of at least 3 behavioural groups, which spend non-nesting months either in the Central Atlantic Ocean, South America, or Southern Africa [19]. Tracked individuals in this study also showed behavioural variation in post-nesting strategy which closely resembles that of the Gabonese population. Two distinct groups were observed to preferentially occupy feeding grounds either in open-ocean habitats along the mid-Atlantic ridge (though seemingly never crossing it), or to display directed movement towards South America. Feeding behaviour could not be ascertained in this study, although displacement curves for all but one individual suggested a potential switch between directed movement away from nesting grounds and more area-constrained movements. Feeding aggregations of leatherback turtles have been observed in Arraial do Cabo in Brazil [64], and at least one turtle in this study (turte 9) showed directed movements towards this region. No turtle was observed to migrate directly towards southern Africa, although these individuals were found to be rare in the Gabon population too [19]. Given the small sample of tagged turtles in this study and the limited temporal coverage of the study that spans a single nesting season, it was therefore not possible to ascertain whether turtles from Bioko also make use of feeding areas in southern Africa.

The threat posed to fishing operations to sea turtles is well established and, in some cases, can be a strong contributor to at sea-mortality [65–67]. Catches of leatherback turtles by vessels targeting tuna or other pelagic teleosts in the region have been documented [68], and areas of particular concern in this respect have been identified for leatherback turtles in the Atlantic Ocean [20]. High levels of fishing threat are estimated to exist in waters surrounding Ascension Island and along the coast of Brazil, which are of interest to the Equatoguinean population as they are located along its migration routes or overlap with likely feeding grounds. The fisheries threat is also likely to be different for the two migratory groups, given the unequal distribution of longlining and purse seining activities. Turtles in the Central Atlantic Ocean migration group are likely to be at higher risk of bycatch, given the overlap of their migratory movements with areas of high activity for longliners near Ascension Island and Saint Helena, as well as less intensive but widespread purse seining activity across the easter central Atlantic Ocean. On the other hand, turtles migrating to South America may experience higher levels of longlining activity as they reach fishing grounds offshore Brazil. The similarity in migration routes between turtles nesting in Bioko and Gabon suggests that they are likely to be similarly exposed to fishing threats.

### Conclusions

The results of this study indicate that Bioko supports important breeding habitat for leatherback turtles that would greatly benefit from an expansion of the existing MPA, which aligns with the government’s intention to improve marine conservation. While this study provides support for an expansion of the marine reserve covering nesting beaches, conservation dividends for the population will only be seen if threats are appropriately managed. Mapping of directed and accidental catches of leatherback turtles within the EEZ should be a priority for conservation in the area, as is the proper enforcement of the marine reserve. For the long-term monitoring and management of the nesting population, trends in nest density, nesting success, and counts of female turtles visiting beaches each season should also be investigated.

Given the global importance of the Gulf of Guinea as a leatherback turtle abundance hotspot, understanding links between nesting groups in the region and uncovering their fine-scale movements should be high priorities for the conservation and management of the species. Furthermore, while no direct connection between leatherback turtles in the study and the Gabonese population was observed, similarities in dispersal patterns are suggestive of existing connectivity between these populations. Further tracking and genetic studies should seek to better elucidate the relationship between these and other central African populations, hence contributing to the development of regional as well as national conservation plans for the species.

Finally, threats to the species during their migration and within feeding grounds should be better quantified by better identifying instances of interaction between animals and fisheries. Refining the spatial resolution of turtle movements would allow for better estimations of these threats, as would data on the vertical use of the water column by both turtles and fishing activities. Linking of spatial and diving data with other data sources, such as accelerometery data for behavioural estimation and sound recordings that may reveal the presence of boats in the vicinity the animals, might further elucidate patterns of interaction and behavioural responses of turtles to fishing boats.

## Supporting information

S1 Table Summary of models chosen for computation of utilisation distribution through ctmm. (PDF)
