# Supplementary material for: Spatial ecology and conservation of leatherback turtles (Dermochelys coriacea) nesting in Bioko, Equatorial Guinea
Source: PLoS One. 2023 Jun 14;18(6):e0286545. doi: 10.1371/journal.pone.0286545 (PMC10266692; doi:10.1371/journal.pone.0286545)
Supplement: S1 Table — (PDF) [file pone.0286545.s001.pdf]

Supplementary Table 1. Summary of models chosen for computation of utilisation distribution through ctmm.

ID	PTT	Model	Period (days)
1	84274	OUF isotropic harmonic 5	10
2	84268	OUF anisotropic harmonic 3	11
3	84269	OUF anisotropic harmonic 0	11.5
4	84272	OUF isotropic harmonic 3	11.5
5	84273	OUF isotropic harmonic 3	15
6	101203	OUF isotropic harmonic 1	14
7	101207	OUF anisotropic harmonic 0	11.5
9	84271	OUF anisotropic circulation harmonic 0	11.5
10	101201	OUF anisotropic harmonic 4	11.5

PTT = Platform Transmitting Terminal
